# Trajectories of body mass index and waist circumference in four Peruvian settings at different level of urbanisation: the CRONICAS Cohort Study

**DOI:** 10.1136/jech-2017-209795

**Published:** 2018-02-22

**Authors:** Rodrigo M Carrillo-Larco, J Jaime Miranda, Robert H Gilman, William Checkley, Liam Smeeth, Antonio Bernabé-Ortiz, CRONICAS Cohort Study Group, Juan P Casas

**Affiliations:** 1 CRONICAS Center of Excellence in Chronic Diseases, Universidad Peruana Cayetano Heredia, Lima, Peru; 2 Department of Medicine, School of Medicine, Universidad Peruana Cayetano Heredia, Lima, Peru; 3 Department of International Health, Bloomberg School of Public Health, Johns Hopkins University, Baltimore, Maryland, USA; 4 Área de Investigación y Desarrollo, AB PRISMA, Lima, Peru; 5 Division of Pulmonary and Critical Care, School of Medicine, Johns Hopkins University, Baltimore, Maryland, USA; 6 Faculty of Epidemiology and Population Health, London School of Hygiene and Tropical Medicine, London, UK

**Keywords:** cardiovascular disease, cohort studies, epidemiology, epidemiology of cardiovascular disease, obesity

## Abstract

**Background:**

Studies have reported the incidence/risk of becoming obese, but few have described the trajectories of body mass index (BMI) and waist circumference (WC) over time, especially in low/middle-income countries. We assessed the trajectories of BMI and WC according to sex in four sites in Peru.

**Methods:**

Data from the population-based CRONICAS Cohort Study were analysed. We fitted a population-averaged model by using generalised estimating equations. The outcomes of interest, with three data points over time, were BMI and WC. The exposure variable was the factorial interaction between time and study site.

**Results:**

At baseline mean age was 55.7 years (SD: 12.7) and 51.6% were women. Mean follow-up time was 2.5 years (SD: 0.4). Over time and across sites, BMI and WC increased linearly. The less urbanised sites showed a faster increase than more urbanised sites, and this was also observed after sex stratification. Overall, the fastest increase was found for WC compared with BMI. Compared with Lima, the fastest increase in WC was in rural Puno (coefficient=0.73, P<0.001), followed by urban Puno (coefficient=0.59, P=0.001) and Tumbes (coefficient=0.22, P=0.088).

**Conclusions:**

There was a linear increase in BMI and WC across study sites, with the greatest increase in less urbanised areas. The ongoing urbanisation process, common to Peru and other low/middle-income countries, is accompanied by different trajectories of increasing obesity-related markers.

## Introduction

Since 1975, the global prevalence of obesity has more than tripled for men and doubled for women.^1^ This scenario is likely to worsen because there would be 573 million obese people in the world by 2030,^2^ and over half of the population in Latin America will be overweight/obese.^3^ The prevalence of obesity in Peruvian adults ranges from 13% in the highlands to 25% in the coastal region.^4^ Also, within Peru, central obesity ranges from 46% in rural Puno to 75% in semiurban Tumbes.^5^ These estimates show a wide variation in obesity indicators where differences exist between rural and urban sites in many low/middle-income countries, an observation that warrants analysing such contexts individually.

Weight and abdominal perimeter change throughout life, with such changes having an effect on health outcomes, including cardiovascular diseases (CVD), mortality and cancer.^6–9^ Studying the trajectory of these obesity-related indicators across different ages will provide a better understanding of how they behave across the lifespan. Trajectory patterns maximise the information available beyond the estimation of incidence rates of overweight or obesity between two assessment points, yet such approaches are not common in countries undergoing economic transitions.

The CRONICAS Cohort Study was conducted in four Peruvian settings with different geographic and socioeconomic characteristics.^10^ These sites, as well as others globally, are at different stages of the nutritional and epidemiological transition^11 12^ with an increasing burden of non-communicable diseases and associated risk factors, including obesity. Thus, we aimed to evaluate the trajectories of body mass index (BMI) and waist circumference (WC) over time according to location in Peru, a middle-income country in Latin America, taking advantage of a combination of rural and urban study sites.

## Methods

### Study design and settings

The CRONICAS Cohort Study is a population-based prospective cohort study conducted in four settings in Peru. The baseline assessment started in September 2010, the first follow-up was conducted, on average, 15 months afterwards, and the second follow-up approximately 30 months after baseline evaluation. Further details about the design of the CRONICAS Cohort Study are available elsewhere.^10^

The four study settings are different in socioeconomic and geographic features: Lima is a highly urbanised metropolis; Tumbes is a semiurban setting in northern Peru; and urban as well as rural Puno are both in the highlands region.^10^ National statistics in 2015 indicated that Puno department, as a whole administrative unit including both rural and urban areas, had a poverty (households where the per capita expenditure is insufficient to purchase basic goods and foods) percentage of 34%–38%, while this figure for Lima and Tumbes ranged from 9% to 12%.^13^ Moreover, health indicators for the year 2013 revealed that 60% of the population in Puno reported being affiliated to any health insurance, 68% in Tumbes and 62% in Lima.^14^

The protocol of the CRONICAS Cohort Study was approved by the Institutional Review Boards at Universidad Peruana Cayetano Heredia and Asociación Benéfica PRISMA in Lima, Peru, and Johns Hopkins University in Baltimore, USA.^10^ Informed consent was obtained from all participants. The research was conducted in accordance with the Helsinki Declaration.

### Study population

A sex and age-stratified (35–44, 45–54, 55–64 and ≥65 years) random sample of participants was identified in each study site, where at least 1000 people were enrolled. Because Puno included both rural and urban settings, 500 participants were enrolled from each location. Exclusion criteria comprised pregnant women, those who could not provide informed consent, were unable to respond to the questionnaires or were bedridden.^10^ Accounting all sites, 11 544 people were identified in a census, 4325 were enrolled and 3741 were surveyed. Overall, at baseline there were 3601 eligible participants with complete interviews. In the first follow-up 2892 (80.3%) were recontacted, and 2726 (75.7%) were recontacted in the second follow-up. For this analysis, the initial sample size at baseline was 3601 participants. Exclusion criteria for the analysis included being younger than 35 years (n=2); in addition, individuals with missing values in sex (n=4) were excluded, resulting in 3595 study members. Furthermore, for the cross-sectional analysis of the baseline data, only participants with complete information in BMI or WC (n=3217; 89.5% of 3595) were included. The number of participants included in each stage of the analysis is depicted in online supplementary figure 1. Online supplementary table 1 shows the comparisons between excluded and included participants at baseline regarding sociodemographic variables and risk factors: there were differences in educational attainment (more people with primary or less education were included), sedentarism (more sedentary participants were included), wealth index (more participants in the top tertile were included) and study site (few participants in Tumbes were excluded).

10.1136/jech-2017-209795.supp1 Supplementary file 1

### Variables

#### Outcome variables

Two were the outcomes of interest: BMI (kg/m^2^) and WC (cm) both treated as continuous variables. These variables were assessed by trained fieldworkers using standardised protocols and equipment across study sites. Height (m) was measured with the participant in the standing position on a flat surface without shoes. Weight (kg) was measured with a TBF-300A body composition analyser (TANITA, Tokyo, Japan). Waist (cm) was assessed with a measuring tape at the middle point between the last rib and the upper limit of the iliac crest.^10^

For descriptive purposes, BMI and WC were categorised at baseline. BMI was divided into underweight (BMI: <18.5), normal weight (BMI: 18.5–24.9), overweight (BMI: 25.0–29.9) and obesity (BMI: ≥30). WC was used to define central obesity according to the International Diabetes Federation WC cut-off points for Ethnic Central and South American populations: ≥90 cm (men) or ≥80 cm (women).^15^

#### Independent variables

Two variables were predictors of interest: study site and time. Time was treated as a categorical variable: 0 was for baseline, 1 for the first follow-up and 2 for the second follow-up round. The main predictor of interest in the regression models was the factorial multiplicative interaction between site and time.

#### Other variables

Other variables assessed with questionnaires at baseline were included in the analysis as potential confounders: educational attainment (less/primary education (<7 years of education), secondary education (7–11 years) and higher (≥12 years)); wealth index, an indicator based on assets and household facilities (in tertiles); physical activity assessed as leisure time and transport-related physical activity according to the International Physical Activity Questionnaire (IPAQ; categories: low, moderate and high);^16^ hours spent watching TV as a proxy of sedentarism (categories:<2 or ≥2 hours/weekday);^17^ heavy drinking defined as having had >1 night of alcohol intake in the previous month and having had ever drunk ≥6 drinks at the same time (categories: yes or no); current smoker, based on the year before the interview (categories: yes or no); and self-reported fruit and vegetable consumption, categorised as <5 or ≥5 servings/day.^18^

### Statistical analysis

All analyses were conducted with STATA V.13.0 (StataCorp, College Station, TX, USA). To describe baseline data, means and SD were used for numeric variables, and proportions (percentages) and 95% CI for categorical variables. Comparisons were performed using the two-sample t-test and Χ^2^ test accordingly. To estimate the outcomes of interest across study rounds, we fitted a population-averaged model by using generalised estimating equations. The dependent variables were BMI and WC, while the independent variable was the factorial interaction between time and study site. The model was specified for Gaussian family and identity link, whereas the correlation structure was set at exchangeable, and the robust option was included.

The analysed model was:

$$Outcome_{ji}=\varphi +\beta_{x}\left(Site_{j}\right)+\beta_{y}\left(Time_{ji}\right)+\beta_{z}\left(Site_{j}\right)\left(Time_{ji}\right)+\beta_{1j}+\varepsilon_{ji}$$

Here ‘*j*’ refers to a given subject and ‘*i*’ to a given time. The outcome could be BMI or WC; φ refers to the intercept of the linear regression; $\beta_{x}$ refers to the regression coefficient for each site; $\beta_{y}$ refers to the regression coefficient of time as a categorical variable; and $\beta_{z}$ refers to coefficient of the multiplicative interaction between site (0=Lima, 1=urban Puno, 2=rural Puno, 3=Tumbes) and time (0=baseline, 1=first follow-up, 2=second follow-up); $\beta_{1j}$ refers to the matrix of covariates (confounders) which were not included as time-dependent variables because they were assessed at baseline; and $\varepsilon_{ji}$ is the SE. The interaction term ($\beta_{z}$) was used to create the figures. We fitted a crude model as well as an adjusted model accounting for educational attainment, wealth index, physical activity, hours spent watching TV, heavy drinking, current smoker, fruit and vegetable consumption and age. The regression models are presented overall and stratified by sex. The adjusted models including covariables were also conducted with the standardised mean of each outcome.

## Results

### Study population at baseline

At baseline, mean age was 55.7 (SD: 12.7) years and the 51.6% of the study members were women. The prevalence of overweight was 43.6% (95% CI 41.9% to 45.4%) and the prevalence of obesity was 26.9% (95% CI 25.4% to 28.5%), while the prevalence of central obesity was 72.7% (95% CI 71.1% to 74.2%). Table 1 shows the characteristics of the study population at baseline according to sex and study site. Across study sites, higher education was more frequent in men than in women, and the same pattern was found for physical activity, current smoking and alcohol consumption. Mean BMI was consistently higher in women than in men, though the opposite pattern was observed for WC.

**Table 1:** Characteristics of the study population at baseline. The CRONICAS Cohort Study

Variables	Lima		Urban Puno		Rural Puno		Tumbes	
	Women	Men	Women	Men	Women	Men	Women	Men
Age	n=542	n=504	n=289	n=274	n=310	n=268	n=519	n=511
Mean (SD)	55.3 (11.9)	55.1 (12.2)	55.9 (12.4)	55.4 (12.6)	56.3 (12.7)	56.9 (13.1)	55.6 (13.0)	56.2 (13.7)
<45	22.5	25.4	24.2	25.2	21.9	22.0	25.6	25.1
45–54	29.0	25.4	23.2	25.6	26.5	24.3	23.9	24.7
55–64	24.7	25.6	26.6	24.8	26.5	24.6	25.8	24.9
≥65	23.8	24.2	26.0	24.5	25.2	29.1	24.7	25.4
Education	n=542	n=503	n=289	n=274	n=310	n=268	n=519	n=510
Primary/less	55.4	29.8	24.2	6.9	78.4	48.5	61.1	50.2
Secondary	29.9	50.1	29.1	24.5	20.3	39.1	27.9	33.8
Higher	14.8	20.1	46.7	68.6	1.3	12.3	11.0	17.1
Wealth index	n=542	n=504	n=289	n=274	n=310	n=268	n=519	n=511
Bottom	15.1	8.7	30.8	16.4	83.9	59.3	36.2	32.9
Middle	39.7	33.7	27.3	25.6	15.2	36.2	38.9	39.1
Top	45.2	57.5	41.9	58.0	1.0	4.5	24.9	28.0
Physical activity	n=542	n=503	n=287	n=274	n=310	n=267	n=519	n=511
Low	24.5	13.5	28.6	13.9	31.9	18.7	70.5	37.8
Moderate	62.9	58.1	61.7	68.6	57.4	66.7	27.2	53.4
High	12.6	28.4	9.8	17.5	10.7	14.6	2.3	8.8
TV watching (hours)	n=542	n=503	n=289	n=274	n=310	n=267	n=519	n=511
<2	56.1	46.9	54.3	53.7	88.7	82.8	49.5	48.7
≥2	43.9	53.1	45.7	46.4	11.3	17.2	50.5	51.3
Current smoker	n=542	n=504	n=289	n=274	n=309	n=268	n=519	n=511
No	95.2	75.6	94.1	84.3	97.7	89.2	97.1	79.3
Yes	4.8	24.5	5.9	15.7	2.3	10.8	2.9	20.7
Heavy drinker	n=542	n=504	n=289	n=274	n=310	n=268	n=519	n=511
No	99.6	88.9	98.3	88.3	98.7	95.2	100.0	88.3
Yes	0.4	11.1	1.7	11.7	1.3	4.9	0.0	11.7
Fruits and vegetables	n=542	n=504	n=289	n=274	n=310	n=266	n=519	n=511
<5/day	91.9	94.8	91.7	94.9	98.4	97.7	98.5	99.0
≥5/day	8.1	5.2	8.3	5.1	1.6	2.3	1.5	1.0
BMI categories	n=542	n=504	n=289	n=274	n=310	n=268	n=519	n=511
Underweight	0.4	0.2	1.0	0.4	1.3	1.5	0.4	0.8
Normal	19.9	26.2	23.9	22.6	45.2	61.9	18.5	29.9
Overweight	39.3	51.2	40.1	59.1	37.7	32.8	42.0	45.4
Obesity	40.4	22.4	35.0	17.9	15.8	3.7	39.1	23.9
BMI	n=542	n=504	n=289	n=274	n=310	n=268	n=519	n=511
Mean (SD)	29.2 (5.0)	27.5 (3.8)	28.5 (5.0)	27.3 (3.4)	25.7 (4.2)	24.4 (3.0)	29.3 (5.0)	27.3 (4.2)
Central obesity	n=542	n=504	n=289	n=274	n=310	n=268	n=519	n=511
No	11.8	38.7	19.7	28.5	42.3	63.4	6.6	29.4
Yes	88.2	61.3	80.3	71.5	57.7	36.6	93.5	70.7
Waist circumference	n=542	n=504	n=289	n=274	n=310	n=268	n=519	n=511
Mean (SD)	91.9 (10.8)	93.0 (10.2)	90.6 (12.0)	95.0 (9.0)	83.0 (12.2)	87.0 (8.9)	94.2 (10.1)	94.9 (10.2)
Height	n=542	n=504	n=289	n=274	n=310	n=268	n=519	n=511
Mean (SD)	148.8 (5.8)	161.0 (6.4)	150.4 (6.0)	163.7 (6.0)	149.8 (5.4)	161.2 (6.1)	151.9 (5.8)	164.7 (6.3)
Weight	n=542	n=504	n=289	n=274	n=310	n=268	n=519	n=511
Mean (SD)	64.8 (11.9)	71.3 (11.7)	64.4 (12.1)	73.1 (10.5)	57.7 (10.7)	63.5 (9.9)	67.7 (12.9)	74.0 (12.8)

Percentages are presented for categorical variables. Regarding numerical variables (BMI, height and weight) the mean (SD) is presented.

BMI, body mass index.

Table 2 shows mean BMI and WC at baseline, and at each follow-up round, according to study site and sex. The mean follow-up time was 2.5 (SD: 0.4) years.

**Table 2:** Variation of BMI and WC by site and sex. The CRONICAS Cohort Study

			BMI	WC	Obesity*
Lima	Baseline	Women	29.2 (5.0)	91.9 (10.9)	40.5 (36.4–44.7)
		Men	27.5 (3.8)	93.0 (10.2)	22.4 (19.0–26.3)
	First FU	Women	29.3 (5.0)	91.8 (10.9)	41.4 (37.3–45.8)
		Men	27.4 (3.9)	93.2 (9.9)	24.5 (20.8–28.6)
	Second FU	Women	29.3 (5.1)	92.1 (11.2)	39.9 (35.6–44.3)
		Men	27.6 (4.0)	93.6 (10.2)	26.7 (22.8–31.1)
Urban Puno	Baseline	Women	28.5 (5.0)	90.6 (12.0)	35.3 (30.0–41.1)
		Men	27.3 (3.4)	95.0 (9.0)	18.0 (13.8–23.0)
	First FU	Women	28.5 (5.0)	92.9 (10.7)	35.0 (28.9–41.7)
		Men	27.3 (3.4)	96.7 (8.4)	17.4 (12.7–23.3)
	Second FU	Women	28.6 (4.9)	91.5 (11.0)	34.0 (27.7–41.0)
		Men	27.7 (3.3)	96.5 (9.3)	19.7 (14.7–25.9)
Rural Puno	Baseline	Women	25.7 (4.2)	83.0 (12.2)	16.1 (12.3–20.7)
		Men	24.4 (3.0)	87.0 (8.9)	3.8 (2.0–6.9)
	First FU	Women	26.2 (4.2)	86.4 (11.0)	16.5 (12.3–21.7)
		Men	24.6 (3.2)	88.7 (9.7)	5.1 (2.9–9.1)
	Second FU	Women	26.7 (4.3)	86.3 (11.4)	22.3 (17.0–28.6)
		Men	24.8 (3.2)	88.1 (9.0)	7.4 (4.4–12.2)
Tumbes	Baseline	Women	29.3 (5.0)	94.3 (10.0)	39.5 (35.3–43.8)
		Men	27.3 (4.3)	95.0 (10.2)	24.2 (20.7–28.1)
	First FU	Women	29.2 (4.9)	93.5 (9.8)	38.1 (33.9–42.6)
		Men	27.3 (4.3)	95.6 (10.0)	22.8 (19.3–26.7)
	Second FU	Women	29.4 (5.0)	94.6 (10.2)	41.3 (36.9–45.8)
		Men	27.6 (4.4)	96.3 (10.2)	24.4 (20.7–28.5)

Results are presented as mean (SD).

*Percentages (95% CI).

BMI, body mass index; FU, follow-up; WC, waist circumference.

### BMI trajectory over time

**Figure 1:**
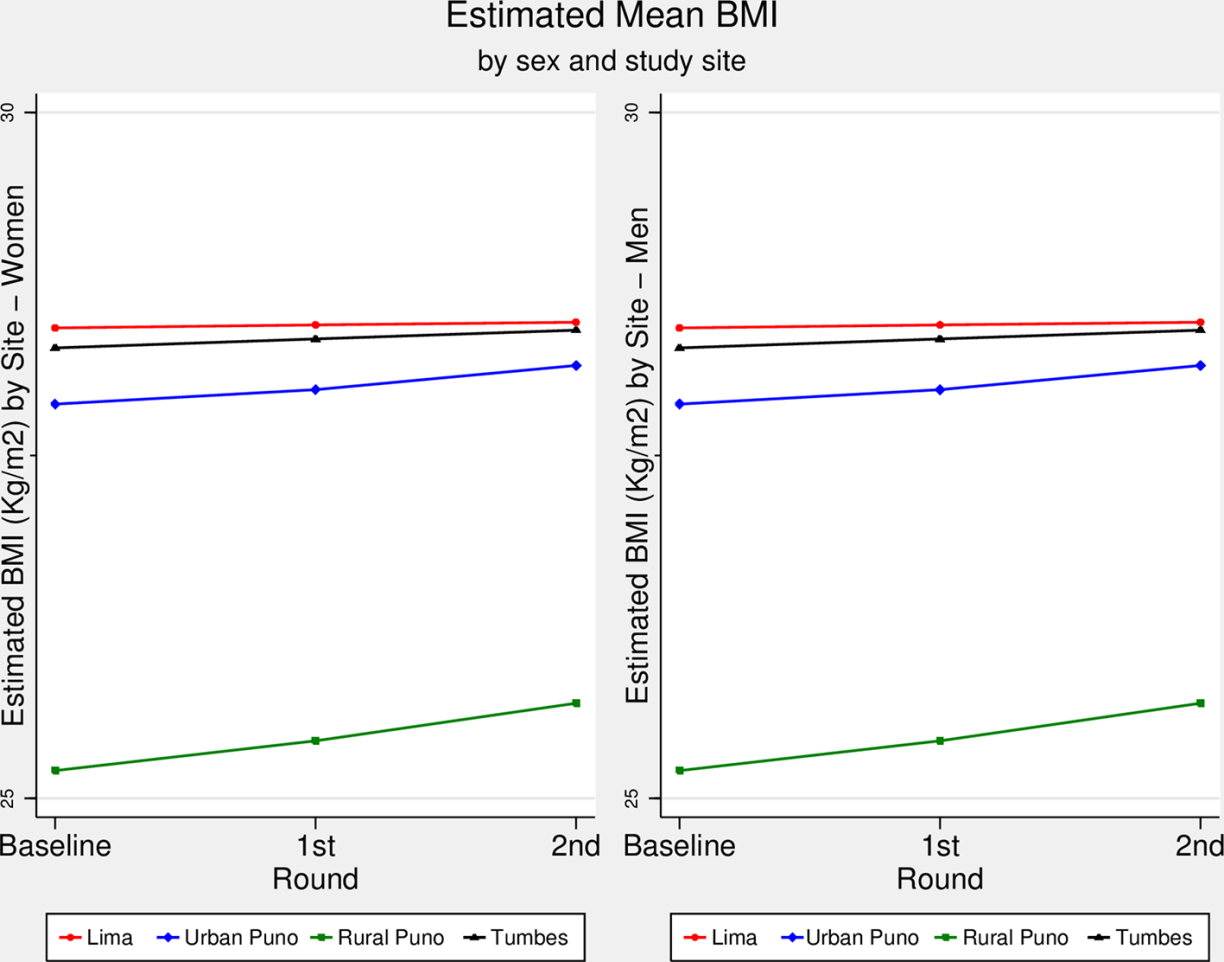
Estimated body mass index (BMI, kg/m^2^) over time by sex and study site. The CRONICAS Cohort Study. The estimated BMI (kg/m^2^) for men and women in each study site and at each follow-up round is shown in this figure. Figure created with the adjusted regression model.

Although at different pace according to the slope of each trajectory, figure 1 depicts that across study sites mean BMI has linearly increased throughout follow-up rounds. Lima showed the highest BMI increase followed by Tumbes, urban Puno and rural Puno. There was strong evidence of different slopes between Lima and urban (P=0.014) and rural (P<0.001) Puno, between urban and rural Puno (P=0.034), and between rural Puno and Tumbes (P<0.001). According to the adjusted overall model and taking Lima as reference (online supplementary table 2), rural Puno showed the fastest increase in BMI (coefficient=0.20, P<0.001), followed by urban Puno (coefficient=0.11, P=0.014) and then Tumbes (coefficient=0.05, P=0.214). A similar trend was retrieved when the results were stratified by sex (online supplementary table 3).

### WC trajectory over time

**Figure 2:**
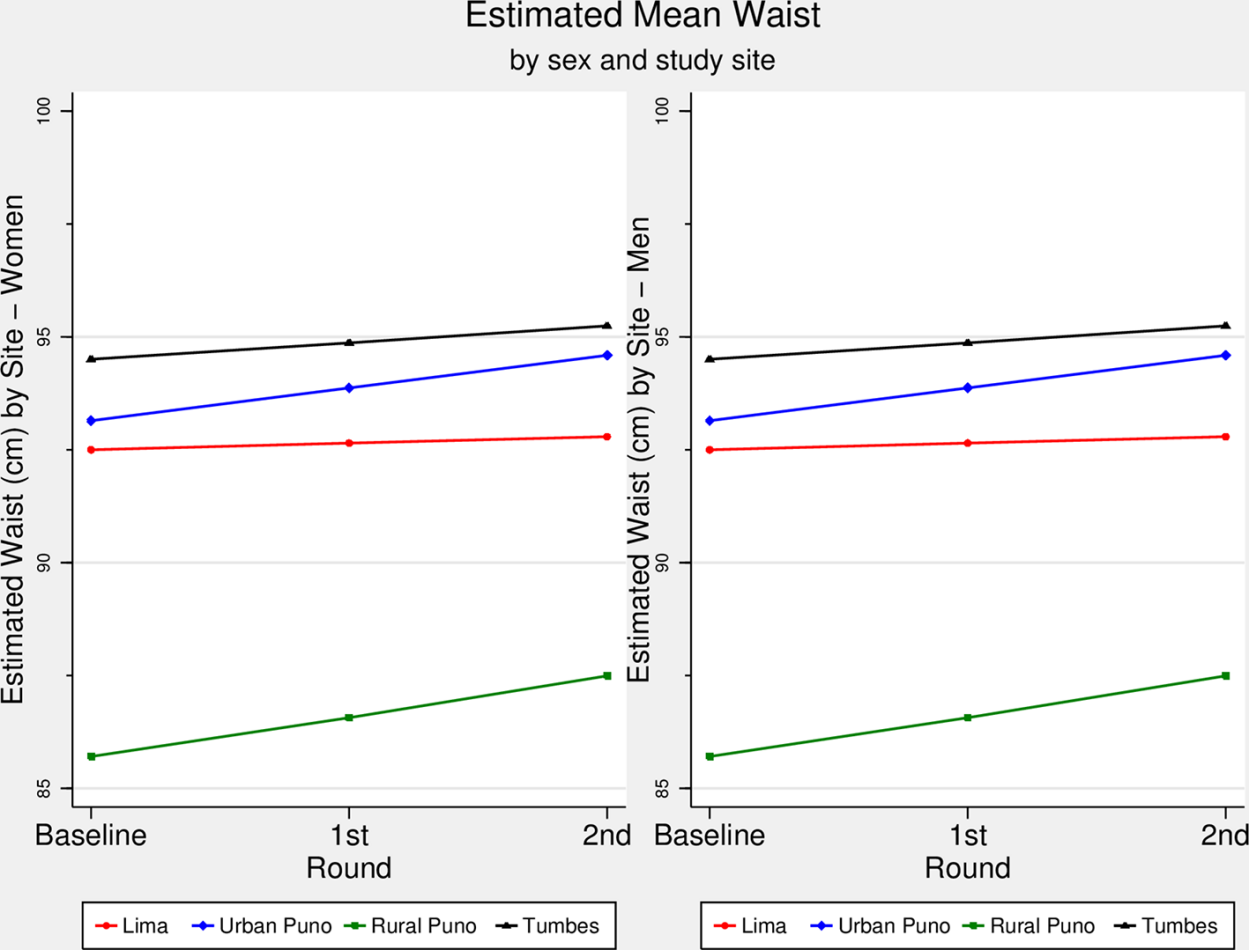
Estimated waist circumference (cm) over time by sex and study site. The CRONICAS Cohort Study. The estimated waist circumference (cm) for men and women in each study site and at each follow-up round is shown in this figure. Figure created with the adjusted regression model.

Figure 2 shows that there was a linear increase in WC across study rounds in each study site, yet according to the slope of each trajectory, the pace of such increase varied among sites. For WC, Tumbes had the highest mean WC across follow-up rounds, followed by urban Puno, Lima and rural Puno (figure 2). The slopes were different between Lima and urban (P=0.001) and rural (P<0.001) Puno, between urban Puno and Tumbes (P=0.033), and between rural Puno and Tumbes (P=0.007). The fastest increase in WC, according to the adjusted model and relative to Lima (online supplementary table 2), was found in rural Puno (coefficient=0.73, P<0.001), followed by urban Puno (coefficient=0.59, P=0.001) and Tumbes (coefficient=0.22, P=0.088). When stratified by sex (online supplementary table 3), a similar trend was found in women, though it was different for men: urban Puno (coefficient=0.42, P=0.061), Tumbes (coefficient=0.29, P=0.080) and rural Puno (coefficient=0.13, P=0.568).

## Discussion

### Main results

Trajectories of BMI and WC increased linearly in all study sites for both men and women. The results showed that in less urbanised settings there was a faster increase in BMI and WC. Relative to Lima, a highly urbanised metropolis, rural Puno had the fastest increase in all adiposity-related outcomes, followed by urban Puno, and then Tumbes. Although the results could have been expected in the current context of weight gain across the globe, this study adds the different trajectories of three adiposity-related markers in sites at different level of urbanisation. This study used repeated measures to estimate BMI and WC trajectories over time, moving beyond static prevalence or incidence rates of overweight/obesity and central obesity. In so doing, this study provides a unique understanding of the complexity of variation, over time and by setting and gender, of adiposity-related markers, thus anticipating scenarios where obesity-oriented interventions are more needed.

### Comparison with other studies

The results show there is a linear increase in BMI and WC. Also, the results depict that rural areas are fattening faster than urban areas. These results were independent of potential confounders including proxy estimates of diet and physical activity. The results are congruent with another study of our group: relative to their rural counterparts, dwellers in urban areas and rural-to-urban migrants have a higher risk of obesity (BMI), yet only the migrant group was at higher risk of central obesity.^19^ In addition, a cross-sectional study in different Peruvian cities concluded that urbanisation is associated with overweight and obesity, particularly in women.^20^ Overall in Peru, it seems that rural dwellers are gaining body fat much faster than their peers in urbanised sites. A possible explanation is the urbanisation process rural sites have been undergoing, which has led to the acquisition of obesogenic profiles.

Similar conclusions have been reached by other authors. In South Africa, a prospective study showed that men and women in rural areas had a greater increase in weight and BMI.^21^ Although they had a longer follow-up time, their annual increase was lower than ours,^21^ suggesting that the obesogenic effect of urbanisation is higher in our sample. Researchers in China studied new cases of obesity between urban and rural sites, finding that obesity incidence was higher in rural than urban settings.^22^ Despite their different analytical approach,^22^ the overall conclusion remains: people in rural sites are at higher risk of having increased adiposity profiles. This is not only a concern of middle-income countries, but high-income countries face the same issue. A cross-sectional study in Sweden showed that WC and BMI were higher in rural than urban sites, with the largest differences in WC (4.8 cm) and BMI (1.8 kg/m^2^) among women.^23^ These figures reveal that people in rural areas deserve further care to help avoid unhealthy weight gain. This is particularly important in low/middle-income countries where rural sites may not have adequate resources to manage the consequences of high cardiovascular risk.

### Results interpretation

The curves presented in the figures are comparable between them and within them. It could be argued that if the starting point of any given outcome is low, as it was the case with rural Puno, there would be more room to observe changes such as gain in weight or other anthropometric indicators. In a post hoc analysis, as an alternative to visualise such effect, we constructed the same figures as those shown in the main results using the standardised mean of each outcome (online supplementary figure 2). These results allow a more direct comparison between the trajectories of interest. The information available in our study prevents us from plotting a diversity of values at the initiation of the observation, hence we are not able to contrast different patterns of increase in trajectories at different starting points. We would need a cohort of people with BMI values at different starting points to compare the pace of change between cohorts in order to conclude whether the starting point is the only, or major, influencing factor for the pace of change.

The increase in adiposity-related indicators could be explained by inadequate patterns of physical activity. Urbanisation comes with less active commuting and less walkable areas, which have been associated with low BMI and weight reduction.^24^ Furthermore, along with transport-related factors, work-related physical activity may also be important.^25^ In rural areas, household chores and agricultural activities, which are still carried out in Tumbes and rural Puno, could be equivalent to moderate/vigorous physical activity;^26 27^ unfortunately, they are being replaced by more sedentary jobs. Because of these inherent characteristics of urbanisation, efforts should be made to make people aware that new lifestyles should also include healthy habits in order to avoid weight and WC gain.^28 29^ Despite these arguments, a study with the same population showed that relative to Lima, urban and rural Puno have less prevalence of sedentarism and transport-related physical inactivity.^30^ Consequently, physical activity may not yet be the main driver of the increased adiposity indexes.

The results could be explained by unhealthy diet patterns and the nutritional transition occurring throughout Peru.^12^ Unfortunately, to the best of our knowledge, there are no reports of diet patterns in the study sites. However, a study in Arequipa (second largest and urbanised city in Peru) found that 42% reported a high-fat diet, 34% low fruit consumption and 33% low vegetable intake.^31^ A population-based study in urban and rural Puno revealed that fat, protein and calories intake was not different between rural and urban areas, although carbohydrate (P=0.03) consumption was slightly higher in the urban group.^32^ These figures suggest that diet profiles in rural Puno, the site with the fastest growth in adiposity indexes herein studied, may be similar to urban counterparts. Interventions to safeguard the healthy characteristics of a rural diet profile among migrant populations and in areas undergoing urbanisation may offer one promising course of action.

Because we did not assess diet and physical activity with gold standard techniques, we cannot hold any of them responsible for our results. Nevertheless, our findings, along with other referenced studies, do signal that the new features of rural sites, regarded as urban characteristics, are playing an important role in the increasing BMI and WC trend.

The fact that BMI and WC changed at different pace across sites calls to assess both—BMI and WC—in clinical evaluations for CVD prevention;^33^ even though some would claim WC is a better parameter regarding CVDs.^34^

### Strengths and limitations

This is a prospective cohort study that has accrued repeated measures of the outcomes of interest. Although the length of the study is short, it allowed enough variation to estimate the trajectories of interest and our results alert of an important ongoing short-term change according to degree of urbanisation, which should be promptly addressed to ameliorate negative health outcomes such as CVDs, cancer and deaths. A recent global study reported that the world age-standardised mean BMI in 1975 was 21.7 kg/m^2^ and 24.2 kg/m^2^ in 2014,^1^ equating to a change of 0.06 kg/m^2^ per year or 0.16 kg/m^2^ over 2.5 years. This figure is lower than the one we have herein reported over a 2.5-year study period, which is 0.30 kg/m^2^ derived from a mean BMI of 27.7 kg/m^2^ at baseline compared with a mean BMI of 28.0 kg/m^2^ at the second follow-up. Therefore, despite the short study period, our results depict an already distressing scenario. Nonetheless, shortcomings of this study should be highlighted. First, the sample included people aged ≥35 years. Having excluded younger individuals could be a limitation, because they could have a different weight change pattern. Yet, the CRONICAS Cohort Study was established primarily to study the risk of CVD among adults, and recruited people aged 35 years and over. Thus, by design, people aged <35 years old were not included as they were considered to be at lower risk of developing cardiometabolic diseases. Second, most covariables were assessed through self-reported methods. While some of the tools used are reliable instruments, such as the IPAQ questionnaire, residual confounding cannot be ruled out. Furthermore, we did not include a more comprehensive assessment of diet profile, relying on a roughly measurement as per fruit/vegetable consumption; also, we did not assess other aspects of physical activity, like work-related physical activity, which could contribute to explain the results of interest. Third, we did not have information on waist-to-hip ratio for the three follow-up rounds, thus it was not included in the study. Future studies should assess this anthropometric parameter, provided it seems to be a better cardiometabolic risk factor than BMI and WC.^35^

## Conclusions

Overall, after 2.5 years of follow-up, there was a linear increase in BMI and WC across study sites. The greatest increase was in the least urbanised areas, relative to the more urbanised ones; similar results were retrieved for men and women independently. This suggests that the ongoing urbanisation process, which is common to Peru and other low/middle-income countries, is accompanied by different trajectories of increasing obesity-related markers.

**What is already known on this subject**

General obesity and abdominal obesity rates are increasing in the world. One of the main drivers of such increase is unhealthy lifestyles, such as sedentarism and poor diet, which are common features in urbanised settings. However, trends of obesity indicators in sites undergoing urbanisation need to be further studied.

**What this study adds**

Trajectories of two obesity indicators—body mass index and waist circumference—in resource-limited settings undergoing urbanisation in a middle-income country in Latin America have steadily increased across the follow-up time. The pace of the trajectories varied according to degree of urbanisation with the fastest increase in less urbanised settings. The trajectories and pace of change in obesity indicators did not differ by sex.
