# Supplementary material for: Trajectories of body mass index and waist circumference in four Peruvian settings at different level of urbanisation: the CRONICAS Cohort Study
Source: J Epidemiol Community Health. 2018 Feb 22;72(5):397–403. doi: 10.1136/jech-2017-209795 (PMC5909748; doi:10.1136/jech-2017-209795)

Trajectories of Body Mass Index and Waist Circumference in Four Peruvian Settings at Different Level of Urbanization: The CRONICAS Cohort Study

Supplementary Material

Supplementary Table 1: Comparison of excluded and included subjects at baseline. The CRONICAS Cohort Study.

	Excluded	Included	p-value
Site (%)	N=378	N=3,217	<0.001
Lima	15.6	32.5	
Urban Puno	52.7	17.5	
Rural Puno	31.2	18.0	
Tumbes	0.5	32.0	
Sex (%)	N=378	N=3, 217	0.766
Female	50.8	51.6	
Male	49.2	48.4	
Age (10-year group, %)	N=376	N=3, 217	0.814
<45	22.1	24.1	
45-54	26.9	25.5	
55-64	26.3	25.4	
≥65	24.7	25.1	
Age (years, mean)	N=376	N=3, 217	0.450
	56.3	55.7	
Education (%)	N=377	N=3, 215	<0.001
Primary or less	41.4	46.2	
Secondary	28.1	32.5	
Higher	30.5	21.3	
Wealth Index (%)	N=378	N=3, 217	<0.001
Bottom	47.6	32.2	
Middle	27.0	33.6	
Top	25.4	34.3	
Physical Activity (%)	N=376	N=3, 213	0.001
Low	22.6	32.0	
Moderate	64.9	55.0	
High	12.5	13.0	
TV Watching (%)	N=377	N=3, 215	0.004
<2 hours	65.3	57.4	
≥2 hours	34.7	42.6	
Current Smoker (%)	N=376	N=3, 216	0.093
No	91.5	88.6	
Yes	8.5	11.4	
Heavy Drinker (%)	N=377	N=3, 217	0.300
No	93.4	94.7	
Yes	6.6	5.4	
Fruits & Vegetables (%)	N=377	N=3, 215	0.023
<5/day	93.4	95.9	
≥5/day	6.6	4.1	

Results are presented as percentages; except for age in years where the mean (standard deviation) is shown. P-values refer to chi^2 test for categorical variables, and to two-sample t-test for numerical variables.

Supplementary Table 2: Regression models for BMI and waist circumference. The CRONICAS Cohort Study.

	Coef. (95% CI)	
	Unadjusted	Adjusted
	Outcome: BMI	
Lima	1	1
Urban Puno	-0.56 (-1.05; -0.07)	-0.09 (-0.62; 0.44)
Rural Puno	-3.23 (-3.67; -2.79)	-2.74 (-3.25; -2.23)
Tumbes	-0.15 (-0.56; 0.26)	0.03 (-0.47; 0.41)
Time	0.02 (-0.03; 0.07)	0.10 (0.04; 0.16)
Urban Puno * Time	0.12 (0.03; 0.20)	0.11 (0.02; 0.20)
Rural Puno * Time	0.22 (0.13; 0.31)	0.20 (0.11; 0.29)
Tumbes * Times	0.05 (-0.03; 0.12)	0.05 (-0.03; 0.12)
Constant	28.43 (28.15; 28.72)	31.66 (30.61; 32.70)
	Outcome: Waist Circumference	
Lima	1	1
Urban Puno	0.64 (-0.53; 1.82)	1.08 (-0.18; 2.34)
Rural Puno	-6.79 (-7.95; -5.63)	-4.84 (-6.12; -3.56)
Tumbes	2.01 (1.10; 2.91)	2.86 (1.87; 3.85)
Time	0.15 (-0.04; 0.34)	0. 14 (-0.05; 0.34)
Urban Puno * Time	0.59 (0.23; 0.95)	0.59 (0.23; 0.95)
Rural Puno * Time	0.73 (0.34; 1.10)	0.73 (0.34; 1.10)
Tumbes * Times	0.22 (-0.03; 0.47)	0.22 (-0.03; 0.47)
Constant	92.50 (91.84; 93.16)	89.25 (86.91; 91.58)

Adjusted model included: education, wealth index, physical activity, hours watching TV, smoking, alcohol consumption, fruits and vegetables consumption and age.

Supplementary Table 3: Adjusted regression model for BMI and waist circumference by sex. The CRONICAS Cohort Study.

	Coef. (95% CI)	
	Women	Men
	Outcome: BMI	
Lima	1	1
Urban Puno	-0.04 (-0.86; 0.78)	-0.26 (-0.88; 0.35)
Rural Puno	-3.08 (-3.86; -2.30)	-2.02 (-2.64; -1.41)
Tumbes	0.09 (-0.58; 0.78)	0.40 (-0.15; 0.94)
Time	0.08 (-0.01; 0.16)	0.09 (0.02; 0.16)
Urban Puno * Time	0.12 (-0.02; 0.26)	0.10 (0.00; 0.20)
Rural Puno * Time	0.29 (0.16; 0.44)	0.10 (-0.00; 0.21)
Tumbes * Times	0.01 (-0.10; 0.11)	0.08 (-0.01; 0.18)
Constant	32.66 (31.13; 34.19)	27.25 (25.86; 28.64)
	Outcome: Waist Circumference	
Lima	1	1
Urban Puno	0.56 (-1.28; 2.40)	1.78 (0.12; 3.44)
Rural Puno	-7.26 (-9.15; -5.36)	-2.78 (-4.47; -1.09)
Tumbes	2.05 (0.62; 3.47)	3.46 (2.08; 4.83)
Time	0.07 (-0.22; 0.37)	0.26 (0.01; 0.50)
Urban Puno * Time	0.74 (0.18; 1.29)	0.42 (-0.02; 0.86)
Rural Puno * Time	1.26 (0.65; 1.86)	0.13 (-0.32; 0.57)
Tumbes * Times	0.13 (-0.24; 0.50)	0.29 (-0.03; 0.62)
Constant	94.74 (91.39; 98.09)	85.59 (82.11; 89.08)

Adjusted model included: education, wealth index, physical activity, hours watching TV, smoking, alcohol consumption, fruits and vegetables consumption and age.

Supplementary Figure 1: Sample size across data analysis procedures. The CRONICAS Cohort Study.

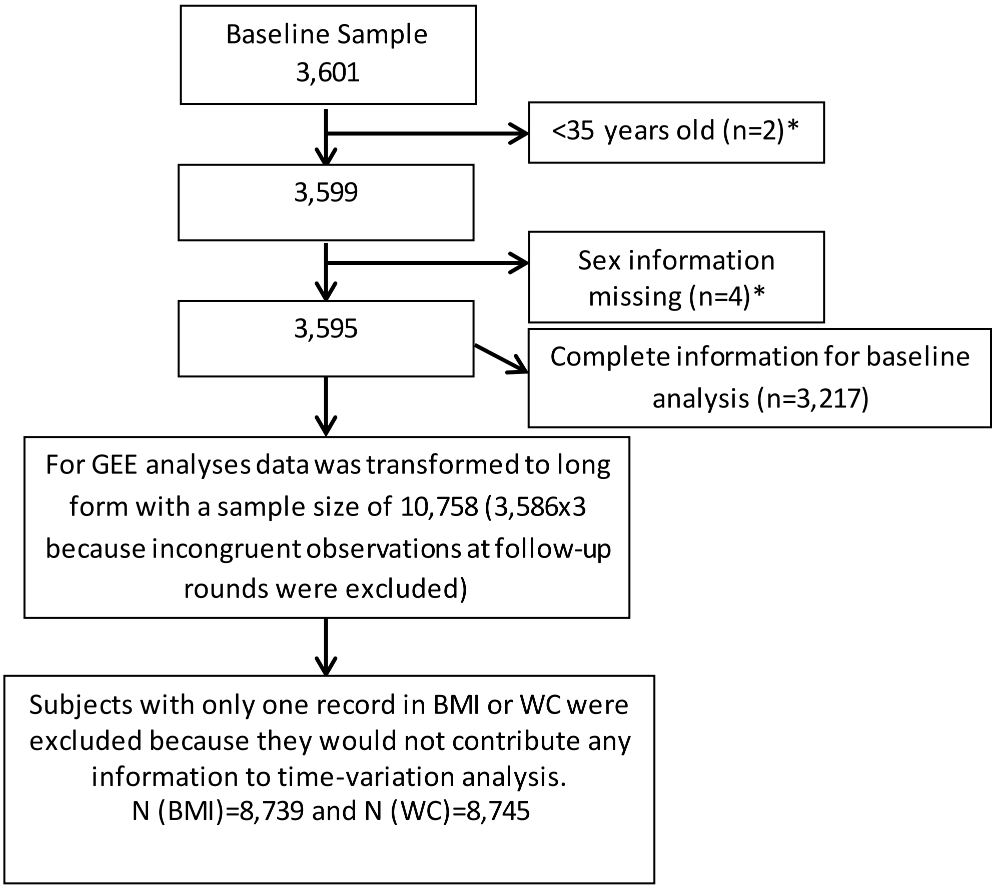

*Observations excluded.

BMI: body mass index; WC: waist circumference.

Supplementary Figure 2: Estimated BMI and waist circumference by sex and study site. The CRONICAS Cohort Study. Outcomes were mean standardized.

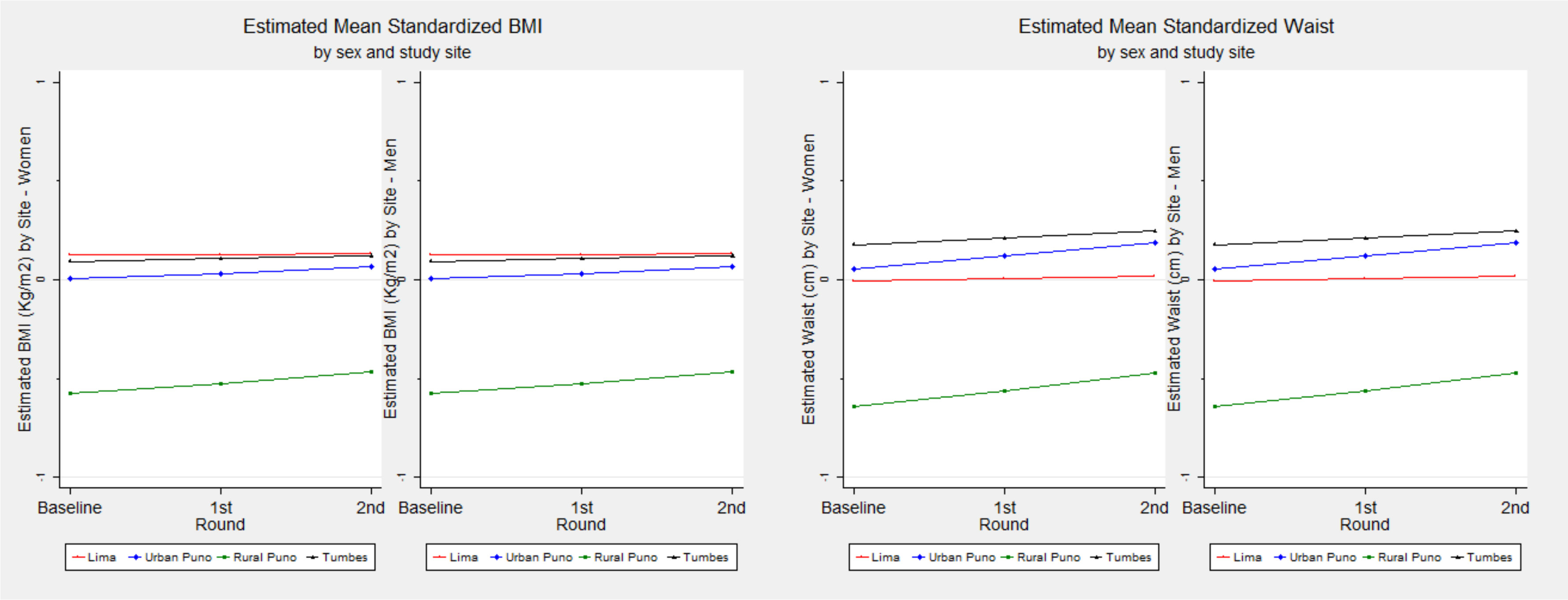

Supplement: Supplementary file 1 [file jech-2017-209795supp001.pdf]
